# Protamine as a barrier against the angiogenic effect of insulin: a possible role of apelin

**DOI:** 10.1038/s41598-023-44639-w

**Published:** 2023-10-12

**Authors:** Mahboobeh Yeganeh-Hajahmadi, Mehrnaz Mehrabani, Mojdeh Esmaili, Mitra Shadkam Farokhi, Mojgan Sanjari

**Affiliations:** 1 Physiology Research Center, Institute of Neuropharmacology, Kerman University of Medical Sciences, Kerman, Iran; 2 Herbal and Traditional Medicines Research Center, Kerman University of Medical Sciences, Kerman, Iran; 3 Cardiovascular Research Center, Institute of Basic and Clinical Physiology Sciences, Kerman University of Medical Sciences, Kerman, Iran; 4 Endocrinology and Metabolism Research Center, Institute of Basic and Clinical Physiology Sciences, Kerman University of Medical Sciences, Boulevard Jahad, Ebne Sina Avenue, Kerman, 76137-53767 Iran

**Keywords:** Endocrinology, Molecular medicine

## Abstract

Insulin is proved to have angiogenic ability thereby may worsen the diabetic retinopathy (DR) progression. Insulin also triggers the expression of endogenous angiogenic peptide, apelin. Since protamine was introduced as an inhibitor of the apelin receptor, we hypothesized that use of protaminated insulin instead of non-protaminated insulin can decrease the negative role of insulin in progression of DR. Firstly, the incidence of DR was compared among three diabetic patient groups: an oral medication, non-protaminated insulin, and protaminated insulin (PIns). Proliferation and migration rate of HUVECs was measured after insulin, apelin, and protamine exposure. In clinical study, the chance of developing DR was 8.5 and 4.1 times higher in insulin group and PIns groups compared with oral group respectively. Insulin group had a chance of 9.5-folds of non-proliferative DR compared to oral group. However, the difference of non-proliferative DR between PIns and oral group wasn’t significant. In-vitro tests showed that concomitant use of insulin and apelin increases viability and migratory potential of HUVECs. However, protamine could reverse this effect. Protamine present in some insulins might show a promising protective role against diabetic retinopathy. Thus, protaminated insulins may be preferable in the treatment of diabetes.

## Introduction

Diabetes mellitus is a chronic and prevalent disease. Based on the international diabetes federation report, its global prevalence in 2019 was about 9.3% (463 million people) and it will rise to 10.2% by 2030^1^.

It has long been believed that microvascular complications in diabetic patients begin several years after the onset of the disease. However, recent studies have shown that the pathological changes that eventually lead to microvascular complications start within a few days to a few weeks of the onset of diabetes^2^. One of these microvascular complications is diabetic retinopathy (DR), which can lead to vision loss. Blindness and vision impairment due to DR increased about two times between 1990 and 2015^3^. The incidence of blindness in diabetics due to retinopathy is 25 times higher in people without diabetes^4^.

DR is classified into two stages. The non-proliferative phase (NPDR) and the advanced or proliferative phase (PDR). NPDR is characterized by changes in the retinal arteries, such as microaneurysms and intraretinal bleeding. PDR is characterized by the formation of new blood vessels in the retina caused by retinal ischemia, followed by an increase in growth factors such as vascular endothelial cell growth factor (VEGF)^5^.

Using insulin is one of the most important ways to treat diabetes. It is needed for all people with type 1 diabetes and may be required in about half of patients with type 2 diabetes^6^. It has been shown that insulin has angiogenic effects^7,8^. Due to the insulin resistance that exists in diabetic patients^9^, higher than physiologic doses of insulin are required to maintain blood sugar within the normal range. This can worsen the angiogenic effects of insulin and causes microvascular complications in diabetic patients.

Apelin is a peptide that binds to a G protein-coupled receptor (GPCR) called APJ^10^. Apelin and APJ are widely expressed in the central nervous system and peripheral tissues including the hypothalamus, adipose tissue, and skeletal muscle^11^. Apelin regulates several physiological functions such as fluid homeostasis, angiogenesis, and energy metabolism^12–14^. It appears that apelin plays a role in promoting DR in people with diabetes. In a clinical study, it was shown that the concentrations of apelin-13 in the vitreous and the fibrovascular retinal membranes of patients with PDR were significantly higher than in the control group^15^. Also, the serum level of Apelin-13 in people with PDR is significantly higher compared to diabetics without PDR^16^. Apelin has been considered as an angiogenic agent in retinal vessels^17^. Apelin administration in vitro induces migration, proliferation, and formation of capillary-like tubules in retinal endothelial cells and stimulates retinal angiogenesis in vivo^17,18^. Furthermore, it was shown that insulin directly control apelin gene expression^19^. Overall, it seems that insulin and apelin have angiogenic effects and this may be one of the mechanisms of diabetic retinopathy in diabetic patients.

Insulin is used both alone and in combination with protamine. Protamine sulfate is a potent alkaline polypeptide commonly used to counteract the effects of heparin. But in combination with insulin it delays insulin absorption and prolongs its effects^20^.

In a study, Gonidec et al. showed that protamine could act as an apelin antagonist and inhibit the effect of apelin on angiogenesis, glucose tolerance, and vasodilation^21^. Thus, protamine that is used in combination with insulins may prevent or delay diabetic retinopathy. On the other hand, protaminated insulins have been used for decades and have been approved by FDA. Therefore, if the beneficial effects of protamine on DR are shown, we can easily prevent or delay diabetic retinopathy by prescribing protaminated insulins instead of insulin alone.

Thus, this study aimed to compare the effect of protamineated insulin (PIns) with insulin on diabetic retinopathy. Since increased angiogenesis is one of the mechanisms of diabetic retinopathy^5^, in-vitro studies on angiogenesis indices in present of insulin, apelin and protamine also was done.

## Materials and methods

The study was done in vitro on diabetic patients and cell cultures. The study was conducted according to the declaration of Helsinki and Good Practice Guidelines and the study protocol was approved by the ethics committee of Kerman University of Medical Sciences, Iran (Permission code: IR.KMU.REC.1398.079). All participants provided written informed consent. The immortalised cell line used in this study were purchased from Pasteur Institute of Iran (Tehran, Iran). Ethical approval for the use of these cells is not required in accordance with local/national guidelines.

### Chemicals

Insulin, protamine sulfate, and cell proliferation reagent WST-1 were purchased from Sigma (Sigma Aldrich, St Louis, MO, USA). Dulbecco's Modified Eagle's Medium-F 12 (DMEM-F12), fetal bovine serum (FBS), Trypsin–EDTA (0.5%), and penicillin/streptomycin were from Gibco (Invitrogen, Carlsbad, CA, USA).

### Human studies

This study was done on diabetic patients that participated in phase 2 KERCADRS (Kerman Coronary Artery Disease Risk Study) which is a study on 10,000 people aged 15–75 years in Kerman—a city in the southeast of Iran—from 2014 to 2018^22^. KERCADRS is a cohort study in which the risk factors of coronary artery diseases are studied. The detailed methodology of this study and the sampling method have been fully described before^23^. In the present study we used cross-sectional baseline data of KERCARS. We included type 2 diabetic patients to our study. Thus, individuals who were available and treated with antidiabetic drugs, diabetes had been previously diagnosed in them, or they had a fasting blood glucose level (FBS) ≥ 126 mg/dl on two consecutive occasions selected and reinvited. We excluded patients with gestational diabetes, a history of exposure to radiation, thalassemia, sickle cell anemia, history of connective tissue diseases such as Behcet's disease, and also patients who were unable to provide informed consent.

200 patients accepted to participate to this study. All participants provided written informed consent. We referred them to an ophthalmologist in order to investigate diabetic retinopathy. A questionnaire was also completed that included information about the type of medication used by them. Other information such as demographic, clinical and experimental information were obtained from KERCADRS. They were divided into three groups based on their medication: Oral medication, insulin and protaminated insulin. Patients who used only oral medication were included in the oral group. Patients who received regular insulin or any of the insulin analogs were included in the insulin group. If they received NPH insulin or insulin analogues containing protamine, they were placed in the protaminated insulin group. Patients who used both oral medications and insulins were excluded from the study (40 patients). 160 diabetic patients were entered the study.

### Cell culture

Human umbilical vein endothelial cells (HUVECs) obtained from Pasteur Institute of Iran (Tehran, Iran) were cultured in DMEM-F12 with 10% FBS and 1% penicillin/streptomycin at 37 °C in a humidified atmosphere of 5% CO_2_.

### Measurement of cell viability

In the viable cells, WST-1 as a type of Tetrazolium salt is cleaved to formazan with the aid of the succinate-tetrazolium reductase system. Hence, the amount of formazan dye formed directly correlates to the number of metabolically active cells in the culture. HUVECs were seeded in 96-well plates at the density of 4000 cells. After 24 h (h), cells underwent FBS free for 24 h. then they were exposed to different concentrations of insulin aspart (20 nM–10 µM), apelin (5–20 nM), protamine (5–1000 µg/ml), and a combination of them at the optimum concentration obtained from previous steps for 48 in growth medium. Afterward, WST-1 reagent for 2 h was added to each well, and absorbance was measured at 450 nm by a microplate reader (BioTek ELX800, Winooski, Vermont, USA). The measured absorbance directly correlates to the number of viable cells.

### Migration scratch assay

HUVECs were plated into 24-wells plates at a density of 2.4 × 10^4^. After 24h, cells had undergone FBS free for 24 h and then treated with an optimum dose of insulin, protamine, and apelin obtained from the previous steps. After 48 h, a scratch was made using a sterilized 1000 μl pipette tip. PBS was used to wash the cellular debris. After adding the growth medium, the migration rate of the scratched area was monitored after 24 h and the pictures were analyzed with NIH Image J software.

### Statistical analysis

The results are reported as mean ± SD. Data with a normal distribution were analyzed using the ANOVA test followed by Tukey post hoc analysis. The relation between diabetic retinopathy with the type of medication was investigated by multinominal logistic regression. Statistical analysis was performed using SPSS 20. All reported P values were two-tailed, and P < 0.05 were considered statistically significant.

### Ethical approval

The study was conducted according to the declaration of Helsinki and Good Practice Guidelines and the study protocol was approved by the ethics committee of Kerman University of Medical Sciences, Iran (Permission code: IR.KMU.REC.1398.079). All participants provided written informed consent. The immortalised cell line used in this study were purchased from Pasteur Institute of Iran (Tehran, Iran). Ethical approval for the use of these cells is not required in accordance with local/national guidelines.

## Results

### Diabetic retinopathy in diabetic patients

Since we saw the inhibitory effects of protamine on angiogenic indices in vitro, we also did a preliminary cross-sectional study on diabetic patients. The demographic data of the studied patients are presented in Table 1. There was no a significant difference in FBS, BMI, HbA1c, and age between the different groups. Diabetes duration in insulin groups was significantly higher than oral group. In addition, there was no significant difference between the groups in terms of blood pressure and blood lipids.

**Table 1:** Demographic and clinical characteristics of diabetic patients based on their medication.

	Oral medication	Insulin	Protaminated insulin
Men	39 (81.3%)	6 (12.5%)	3 (6.3%)
Women	93 (83.0%)	10 (8.9%)	9 (8.0%)
Age	58.19 ± 8.44	53.13 ± 11.89	57.67 ± 10.90
BMI (kg/m^2^)	28.12 ± 4.1	30.89 ± 6.39	30.40 ± 5.70
FBS (mg/dl)	169.55 ± 57.21	186.13 ± 80.66	202.67 ± 72.07
HbA1c (%)	8.26 ± 1.77	8.39 ± 1.31	9.08 ± 1.85
Diabetes duration (months)	79.44 ± 71.17	145.75 ± 90.64*	102.25 ± 93.46
Hypertension	75 (78.9%)	11 (11.6%)	9 (9.5%)
Dyslipidemia	125 (83.9%)	14 (9.4%)	10 (6.7%)
Insulin therapy duration (months)		86.8 ± 66.7	80.1 ± 86.7

BMI: body mass index, FBS: fasting blood sugar, HbA1c: hemoglobin A1c.

**P < 0.05 vs. oral medication group. Data presented as mean ± SD and n (%).

To investigate any association between type of medication and diabetic retinopathy in the first step, we did binary logistic regression for each independent variable. Then we included independent variables with P ≤ 0.2 (age, BMI, diabetes duration, HbA1C, and hypertension) into final model and did multinominal logistic regression. Crude odds ratios obtained from logistic regression are shown in Table 2. Multinominal logistic regression showed that insulin treatment increases the chance diabetic retinopathy 8.5-folds (P = 0.003) in comparison to oral medication. However, this chance was 4.1-folds in protaminated insulin group (P = 0.044). In the next step we subdivided the diabetic retinopathy into proliferative and non-proliferative. The chance of developing non-proliferative retinopathy was about 9.5-folds in insulin group in comparison to oral medications (p = 0.004). However, the change in this chance was not significant in protaminated insulin group (Table 3). The chance of proliferative retinopathy in diabetic patients who had received insulin and protaminated insulin wasn’t significantly different from oral group. (Table 3).

**Table 2:** Crude odds ratios from binary logistic regression for all independent variables.

Independent variable	Regression coefficient (B)	SE	P-value	Crude odds ratio (CI)
Age	0.062	0.021	0.004	1.064 (1.020–1.110)
BMI	− 0.060	0.039	0.125	0.942 (0.872–1.017)
Sex	− 0.015	0.377	0.969	0.985 (0.470–2.064)
FBS	0.003	0.003	0.355	1.003 (0.997–1.008)
Diabetes duration	0.005	0.002	0.046	1.005 (1.000–1.009)
HbA1C	0.219	0.103	0.033	1.245 (1.018–1.523)
Dyslipidemia	0.658	0.754	0.383	1.930 (0.441–8.458)
Hypertension	0.906	0.364	0.013	2.475 (1.212–5.054)

BMI: body mass index, FBS: fasting blood sugar, HbA1c: hemoglobin A1c.

**Table 3:** Multinuminal logistic regression of diabetic retinopathy with the type of medication.

Variable		Regression coefficient (B)	SE	P-value	Adjusted odds ratio	95% CI
Dependent	Independent					
Diabetic retinopathy	Age	0.060	0.025	0.019	1.061*	1.010–1.115
	BMI	− 0.068	0.046	0.138	0.934	0.853–1.022
	Diabetes duration	0.001	0.003	0.634	1.001	0.996–1.007
	HbA1c	0.215	0.114	0.059	1.240	0.992–1.552
	Hypertension	0.675	0.429	0.116	1.964	0.847–4.555
	Protaminated insulin	1.411	0.700	0.044	4.101*	1.041–16.165
	Insulin	2.137	0.726	0.003	8.473*	2.041–35.185
Non-proliferative diabetic retinopathy	Age	0.080	0.028	0.005	1.083*	1.024–1.145
	BMI	− 0.069	0.051	0.177	0.933	0.845–1.032
	Diabetes duration	0.001	0.003	0.716	1.001	0.995–1.007
	HbA1c	0.243	0.124	0.050	1.275*	1.000–1.625
	Hypertension	0.702	0.463	0.129	2.017	0.815–4.995
	Protaminated insulin	1.247	0.764	0.103	3.480	0.778–15.561
	Insulin	2.252	0.776	0.004	9.503*	2.076–43.490
Proliferative diabetic retinopathy	Age	− 0.001	0.042	0.980	0.999	0.920–1.084
	BMI	− 0.066	0.077	0.392	0.936	0.805–1.089
	Diabetes duration	0.002	0.005	0.653	1.002	0.993–1.011
	HbA1c	0.122	0.192	0.525	1.130	0.775–1.648
	Hypertension	0.604	0.772	0.434	1.829	0.402–8.310
	Protaminated insulin	1.720	0.971	0.076	5.584	0.833–37.424
	Insulin	1.686	1.109	0.128	5.400	0.615–47.427

HbA1c: hemoglobin A1c. BMI: body mass index *P < 0.05.

### Cell cultures

#### Measurement of cell viability

**Figure 1:**
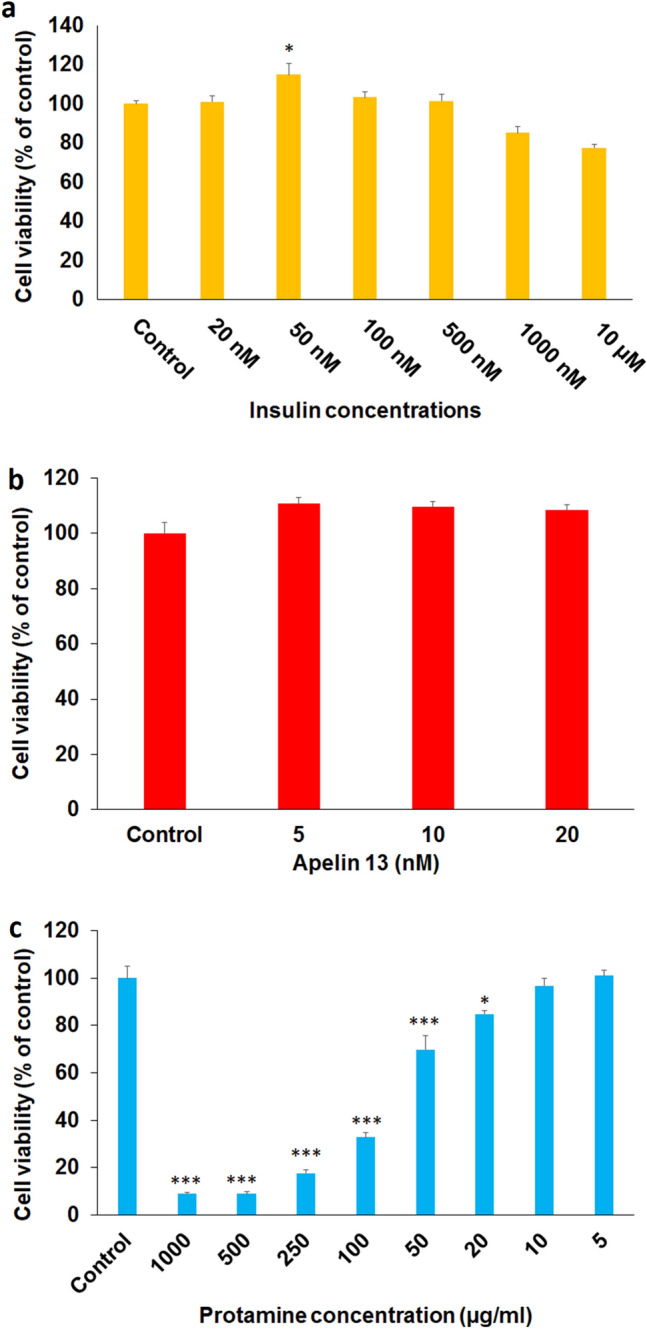
Dose–response data related to the effects of different doses of insulin aspart (**a**), Apelin (**b**), and Protamine (**c**) on cell viability. Cells were treated with various concentrations of insulin aspart, apelin, or protamine. Cell viability was estimated using WST-1 assay. *P < 0.05, ***P < 0.001 vs control.

Insulin was not able to increase cell viability after 24 h (data was not shown). However, after 48 h, insulin at a concentration of 50 nM increased the viability of HUVECs (Fig. 1a).

As shown in Fig. 1b, apelin at a concentration of 5, 10, and 20 (based on the previous study) did not alter the viability of cells after 48h.

Protamine was toxic for cells in doses higher or equal to 20 µg/ml after 48h (Fig. 1c).

**Figure 2:**
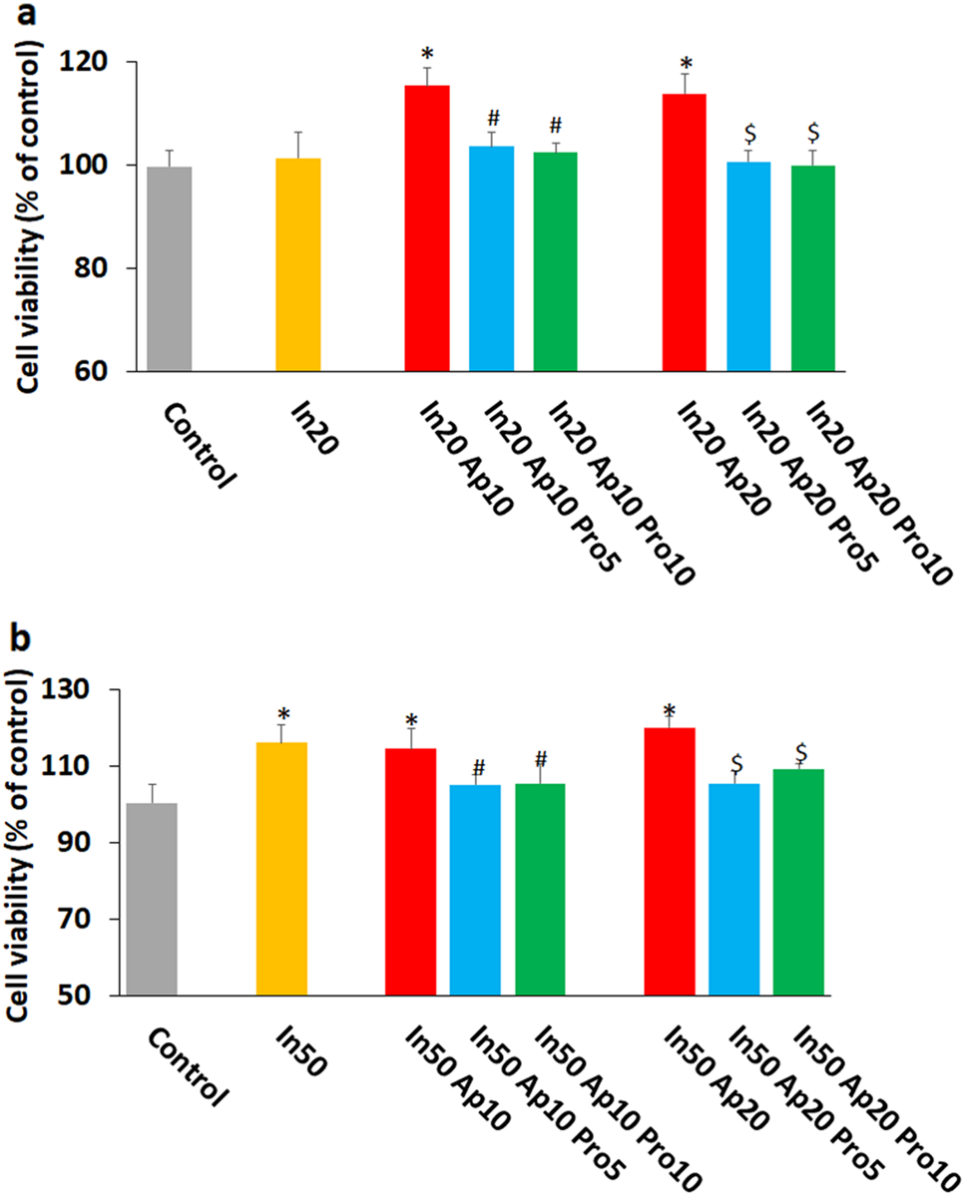
Effect of combination of insulin, apelin, and protamine on HUVECs viability. The combination of insulin 20 nM (**a**) and 50 nM (**b**) with apelin 10 and 20 nM markedly increases the viability of HUVECs. This effect was inhibited by protamine at concentrations of 5 and 10 µg/ml. In 20: Insulin 20 nM, In 50: Insulin 50 nM, Ap 10: Apelin 10 nM, Ap 20: Apelin 20 nM, Pro 5: Protamine 5 µg/ml, Pro 10: Protamine 10 µg/ml. *P < 0.05 vs control, ^#^P < 0.05, vs In (20 or 50) Ap10, ^$^P < 0.05 vs In (20 or 50) Ap20.

The combination of insulin 20 nM with apelin 10 and 20 nM markedly increases the viability of HUVECs. This effect was suppressed by protamine at a non-toxic concentration of 5 and 10 µg/ml (Fig. 2a). This was true also for insulin 50 nM (Fig. 2b).

#### Migration scratch assay

**Figure 3:**
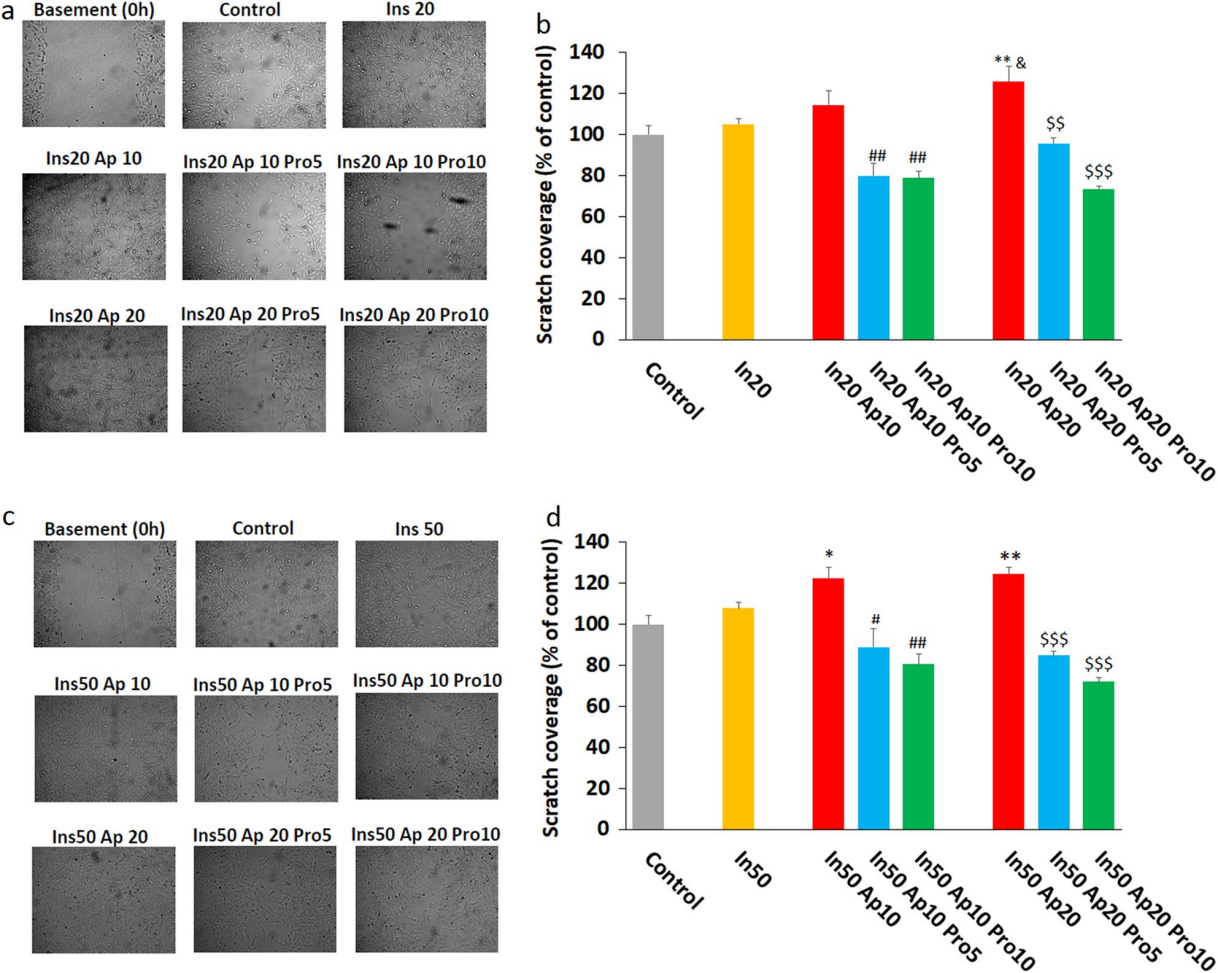
Effect of combination of insulin, apelin, and protamine on HUVECs migration. The combination of insulin 20 nM (**a** and **b**) and 50 nM (**c** and **d**) with apelin 10 and 20 nM markedly increases the migration of HUVECs. This effect was inhibited by protamine at concentrations of 5 and 10 µg/ml. In 20: Insulin 20 nM, In 50: Insulin 50 nM, Ap 10: Apelin 10 nM, Ap 20: Apelin 20 nM, Pro 5: Protamine 5 µg/ml, Pro 10: Protamine 10 µg/ml. *P < 0.05 vs control, ^#^P < 0.05, ^##^P < 0.01 vs In (20 or 50) Ap10, ^$^P < 0.05, ^$$^P < 0.01, ^$$$^P < 0.001 vs In (20 or 50) Ap20.

The migration assay showed that insulin increases the migration of cells somewhat. However, it was not significant. Combinations of insulin 20/apelin 20 and insulin 50/apelin 10 and insulin 50/apelin20 were able to increase the migratory potential of cells. This effect was suppressed by protamine at concentrations of 5 and 10 µg/ml (Fig. 3).

## Discussion

In this study, we showed that in diabetic patients who had received insulin the chance of diabetic retinopathy was about sixfolds in comparison to oral medication group. However, this chance was about fourfolds in protaminated insulin group. Furthermore, the chance of developing non-proliferative retinopathy in insulin group was 5.3-folds (P = 0.012) But it wasn’t significant in protaminated insulin group. the chance of proliferative retinopathy in both insulin and protaminated groups was significantly more than oral medication group. In addition, in-vitro studies showed that the concomitant use of insulin and apelin increases the survival and migration of HUVEC cells (in vitro angiogenesis indices). But protamine inhibits these effects. Therefore, it seems that protamine can play a protective role against diabetic retinopathy.

The pathophysiology of DR is complex and there are several treatments due to this complexity. Unfortunately, none of the treatments have been able to completely control the progression of the disease or reverse the damage to the retina. In addition, some treatments are associated with side effects. Therefore, finding new treatments for DR as well as preventing its occurrence is very important^24^.

The progression of diabetic retinopathy is closely related to the pathological angiogenesis of the retina. Therefore, one of the treatments for retinopathy is to prevent the formation of new blood vessels. For example, anti-VEGF drugs are being used as one of the main drugs in retinopathy. These drugs have beneficial effects on some patients. But most patients have achieved little clinical improvement^25^. In addition, these drugs have a short half-life, so monthly or twice-monthly injections are essential. Therefore, the acceptance of these drugs by patients is low^25^.

Insulin, and apelin have been shown to have angiogenic effects and exert this effect independently of VEGF^7,8,15,16^. Therefore, inhibition of angiogenic effects of these two peptides can be considered as an important goal in the prevention or treatment of diabetic retinopathy.

Insulin is the most important treatment for diabetes, so all people with type 1 diabetes and half of the patients with type 2 diabetes should use insulin. Unfortunately, due to insulin resistance in these patients, high doses of insulin are needed to lower blood sugar. Also, in persons with diabetes who are at the beginning of the disease and don’t need insulin injection, endogenous insulin increases as a compensatory mechanism^26^. Insulin appears to be a double-edged sword. On the one hand, glycemic control can prevent angiogenesis and thus the progression of diabetic retinopathy, but on the other hand, it has angiogenic effects per se^7,8^. In addition, several studies have shown that apelin levels increase in the plasma and vitreous of persons with diabetes^15,16^. Therefore, it seems that high levels of insulin and apelin are present in the vitreous of these patients. Furthermore, it was shown that high insulin levels are associated with high levels of apelin and insulin directly control apelin gene expression^19^.

In this study we showed that we showed that the chance of developing diabetic retinopathy in people receiving the PIns was fourfolds more than oral treatment group, but it was about sixfolds in insulin group. In addition, while the chance of non-proliferative retinopathy in the insulin group had a significant difference compared to the oral treatment group, it was not significant in the protaminated insulin group. At first, we thought that insulin could increase angiogenesis by increasing apelin. But in in-vitro experiments, we observed that apelin alone is not able to increase angiogenesis, however in the presence of insulin, it enhances the angiogenic effects of insulin. This effect can be inhibited by protamine. Given that it has been shown that protamine can act as a selective antagonist of the apelin receptor^21^, it is probable that protamine plays a protective role against retinopathy via inhibiting the apelin receptors.

Only a few studies have compared the effect of protaminated insulin with non-protaminated. The results obtained in these studies were not consistent^27–30^. Our study is in agreement with Rosenstock et al. study in which they reported that diabetic retinopathy is 7% in glargine^30^ But it was 2.7% in NPH group^30^. However, Lin et al. showed that glargine and NPH insulins have similar effects on diabetic retinopathy^28^.

It has been shown that hyperglycemia and hyperinsulinemia in diabetic patients can increase the risk of cancer. Therefore, the use of protaminated insulins may not only prevent the progression of retinopathy but also prevent cancer in these people. The anti-carcinogenic effects of protamine have also been shown in other studies^31,32^.

One of the limitations of this study was the small number of participants in the human study part. In this study, we used KERCADR cohort samples. This cohort examines the risk factors of coronary artery diseases. The prevalence of diabetes in this study is reported to be about 10%^33^. We re-invited all those who were available and had diabetes. We were able to recruit this number. But as said, this is a preliminary study that can be the basis for further studies. Another limitation of our study was that we did not have access to family history of retinopathy and hypoglycemic episodes. Thus, larger studies taking into account all risk factors would be very helpful. In addition, long-term prospective studies on different types of insulin are recommended.

## Conclusion

The use of protamine was able to inhibit apelin and insulin-induced angiogenesis. In addition, the chance of diabetic retinopathy in protaminated insulin group is less than insulin group. Therefore, the use of protaminated insulins in diabetic patients seems to be a better option than non-protaminated insulins.

## Data Availability

The datasets used and/or analyzed during the current study are available from the corresponding author on reasonable request.

## Abbreviations

DR: Diabetic retinopathy
FBS: Fasting blood sugar
GPCR: G protein-coupled receptor
HbA1c: Hemoglobin A1c
HUVEC: Human umbilical cord endothelial cells
NPDR: Non-proliferative diabetic retinopathy
PDR: Proliferative diabetic retinopathy
PIns: Protaminated insulin
VEGF: Vascular endothelial cell growth factor
